# A 17-Year Experience of a Large Dedicated Fellowship in Blood and Marrow Transplantation and Cellular Therapy: A Blueprint for Modern Day Training Program

**DOI:** 10.1007/s13187-024-02545-3

**Published:** 2024-11-27

**Authors:** Sushma Bharadwaj, Robert Lowsky, Lekha Mikkilineni, Melody Smith, Wen-Kai Weng

**Affiliations:** Division of Blood and Marrow Transplantation and Cellular Therapy, Stanford University School of Medicine, Stanford, CA94304 USA

**Keywords:** Blood and marrow transplantation, Cell therapy, Fellowship, Training

## Abstract

The field of allogeneic blood and marrow transplantation-cellular therapy (BMT-CT) has evolved through incremental advances. Engineered donor grafts, gene editing and chimeric antigen receptor T-cells are all standard clinical practice. Consequently, the scientific knowledge and complexity of clinical skills needed for next generation of BMT-CT physicians have increased. We report a 17-year experience of arguably the largest 12-month BMT-CT clinical fellowship program in the USA. Seventy-three (73) trainees were accepted and 2 cohorts that reflected different time periods (2007–1016 and 2017–2024, inclusive) and different core training curriculum were compared. The cohorts were equivalent in terms of demographics; notably, most (70%) had graduated from international medical schools and trained in the US on a non-immigrant J1 visa. In 2015, we introduced a structured mentoring program to address the desire of trainees for experience with scholarly activities. There was a high rate of successful academic careers with a trend toward a higher likelihood of academic retention following structured mentoring (70% vs 89%). In this report, we included our detailed core curriculum and highlight potential future changes as a blueprint for modern day programs to ensure that graduate “transplant docs” can continue to contribute at the highest academic level.

**Supplementary Information:**

The online version contains supplementary material available at 10.1007/s13187-024-02545-3.

## Introduction

The field of hematopoietic cell transplantation (HCT) has evolved from its early beginnings in the 1940s to a present-day standard of care potentially curative therapy for patients with high-risk hematologic malignant diseases and bone marrow failure syndromes. By 2019, it was estimated that more than 1.5 million worldwide patients received a blood or marrow transplant in one of the 1500 transplant centers [1]. Whereas initially survival was measured in days after transplant, patients with otherwise fatal cancers now attain long-term disease-free survival and cure and reclaim their personal, social, and working lives many of who are free of transplant-associated complications. The recent translation of engineered grafts, immune effector cells (IEC), and genetically modified cells to clinical practice marks yet another significant evolution and defines next-generation cellular therapies for cancer as well as nonmalignant diseases.

For more than two decades, a number of US academic centers developed a 6 to 12 months blood and marrow transplant (BMT) clinical fellowship designed to prepare post-hematology-oncology trainee physicians for academic careers focused on transplantation and cellular therapy. The American Society for Blood and Marrow Transplantation (ASBMT) published initial guidelines that outlined the cognitive and procedural skills needed for trainees to provide optimal care for HCT recipients [2, 3] and later developed a detailed BMT training curriculum based on sound educational principles [4]. More recently, the American Society for Transplantation and Cellular Therapy (ASTCT) developed guidelines to standardize fellowship training in HCT and IEC [5]. In the short period since this latter publication, our field has continued to evolve at a rapid rate including novel cellular therapies extending to non-malignant diseases [6, 7]. Consequently, academic HCT programs face a unique and exciting challenge, part of which is to develop a training curriculum that is adapted to the changing paradigms in treatments, so current and next-generation cellular therapists provide the highest quality of patient care.

Here, we report the experience of arguably the largest dedicated BMT fellowship training program in the USA and detailed analysis of the demographic changes of the fellows, career positions throughout time, and the impact of the structured mentoring program on the scholarly accomplishments. Finally, we included our detailed core curriculum as a blueprint for modern day training program.

## Methods

### Institutional Setting

Stanford is a large research university with hospitals, seven schools of study [Humanities and Sciences, Law, Medicine, Business, Earth Sciences, Engineering, and Education] and more than 35 Centers and Institutions all located on the same campus providing excellent facilities for scientific research and education. Within the School of Medicine, the adult and pediatric BMT-CT programs are independent from one another, located in different hospitals and with no shared overlapping clinical faculty. The Adult Division of BMT-CT is also separate from the Divisions of Adult Hematology and Oncology. The BMT-CT division is a high-volume transplant center with abundant resources for training next-generation BMT-CT specialists. There are 18 full-time faculty members (10 female and 8 male) and 6 research laboratories within the Division.

### BMT-CT Fellowship

In 1994, our institution started a 6-month adult clinical BMT fellowship and by 2004 had trained only 12 fellows. We expanded the training to 12 months in 2004, and we created and established an adult BMT Fellowship Core Training Curriculum and national advertisements to solicit applicants in 2007 (Supplement 1). We shared our curriculum with Dr. Linda Burns in 2010 (personal communication, RL) that was used in part as a template for the ASBMT guidelines for fellowship training in BMT in 2012 [4]. Over time, we expanded our fellowship program to consist of 5–7 clinical BMT-Cellular Therapy (BMT-CT) Fellows from July 1, of 1 year, to June 30 the following year. In 2015, we established and adopted a structured mentoring program as a method to enhance scholarly productivity among our fellows (Supplement 2). Curricula training in CAR-T therapy was added in 2019, and curricula related to immune effector cell therapy for non-malignant diseases is planned to be added for the incoming year in 2025 (see discussion). The current positions of our fellowship graduates were updated as of August 1, 2024. Cohort 1 was defined as Fellows during the 10-year period (first decade) from July 1, 2007, until June 30, 2017, inclusive. Cohort 2 composed of Fellows from July 1, 2017, to completion of June 30, 2024.

### Academic Productivity

Academic productivity was defined as any authored or co-authored full-length, peer-reviewed research publication or review article that was initiated during fellowship and published within 24 month upon completion. For this analysis, the time periods for comparison were academic productivity in the 8 years prior to implementation of the structured mentoring plan (July 1, 2007–June 30, 2015) to the 7-year period after the mentoring planned was implemented (July 1, 2015–June 30, 2022). This allowed the latter group to fulfill the defined 24-month follow-up.

### Statistics

Descriptive statistical analyses and a two-tailed Fisher’s exact test to determine difference in outcomes between the two cohorts were performed with PRISM (GraphPad, San Diego, CA). For academic productivity, the two time periods are compared as an incidence ratio t-test. The incidence ratio is computed as the number of events (publication) initiated during the year of fellowship over the denominator defined as person-years.

## Results

### Division Clinical Activity

**Fig. 1:**
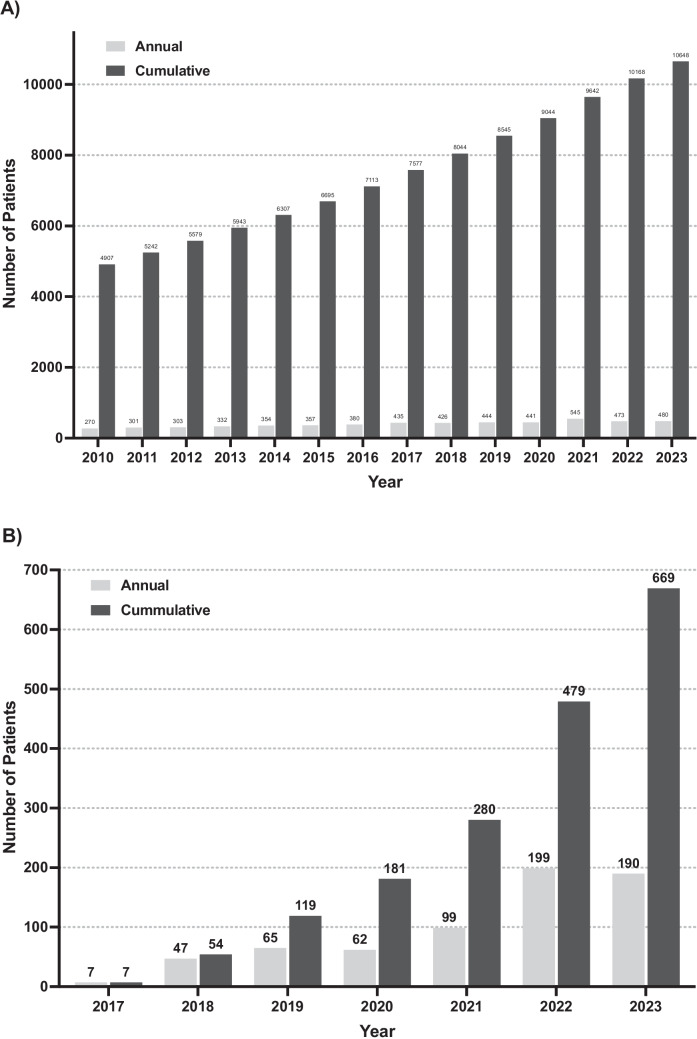
Annual and cumulative number of adult patients who received blood and marrow transplant (**a**) or CAR-T/cell therapy (**b**) at Stanford

Our adult BMT-CT Program has experienced significant growth (Fig. 1a and b). The San Francisco Bay area has a population of close to 8 million and is served by only two active transplant programs (Stanford and UCSF); consequently, there is a robust number of referrals and from socioeconomically diversified patient populations. In FY23, the adult BMT-CT program performed 238 autologous HCT, 252 allogeneic HCT, and 190 CAR-T therapies.

### Fellow Characteristics

The demographics and characteristics of the fellows are highlighted in Table 1. From July 1, 2007, to June 30, 2024, inclusive 73 fellows were accepted to the division’s BMT-CT fellowship. Seventy fellows completed a 2 or 3-year Hematology and/or Medical Oncology fellowship program prior to the start of their BMT fellowship. Three fellows joined our BMT fellowship program directly after completing an Internal Medicine residency program. Seventy-two (72) trainees successfully completed their 12-month fellowship, and one fellow withdrew from the program 4-months into the fellowship.

**Table 1:** Fellow characteristics

Characteristics	Total *n* = 73	Cohort 1 (2007–2017) *n* = 34	Cohort 2 (2017–2024) *n* = 39
Sex, no. (%)			
Female Male	18 (25) 55 (75)	9 (26) 25 (74)	9 (23) 30 (77)
Professional certificate			
MD DO	71 2	33 1	38 1
Race			
Arab Asian Black East Asian Han Latino Caucasian	5 34 2 4 4 6 18	2 18 0 2 2 1 9	3 16 2 2 2 5 9
Medical degree			
AMG IMG India Pakistan South American Canada Caribbean Middle Ease Southeast Asia Australia Nigeria	22 51 22 5 6 4 4 4 4 1 1	13 21 12 3 1 0 0 2 2 1 0	9 30 10 2 5 4 4 2 2 0 1
Careers (*n* = 72)			
Academic Group Medical Practice Industry	59 11 2	24 7 2	35 4 0

*MD* doctor of medicine, *DO* doctor of osteopathic medicine, *AMG* American medical graduate, *IMG* international medical graduate

Of the 73 fellows that were accepted, 18 were female, 1 of whom was also Latino. Of the 55 male fellows, 7 were under-represented minorities (5 were Latino and 2 were Black/African American). There was no difference in the representation of women in cohort 1 (the first decade from 2007 to 2017, inclusive) compared with cohort 2 from July 1, 2017, onwards (26% vs 23%, p = 0.790). There was a trend towards more under-represented minority fellows accepted in cohort 2 (*n* = 7, 18%) compared with cohort 1 (*n* = 1, 3%), yet this did not achieve significance, *p* = 0.060. The majority of fellows that were accepted to our program were international medical graduates (IMG) (51/73, 70%) and most were on a non-immigrant exchange program J1 Visa (46/51 = 90%). Three US citizens were part of the IMG group. The ratio of IMG did not significantly differ in comparing cohort 1 to cohort 2 (61% vs 77%, *p* = 0.204).

The most common countries of origin for medical school were the USA (n = 23) and India (n = 22), and thereafter the next cluster was much lower in number with graduates from Pakistan (*n* = 5) and the combined South American countries of Peru, Venezuela, Columbia, and Argentina (*n* = 6). From among the 50 IMG that completed their BMT-CT fellowship, only 6 returned to their country of origin; the 4 Canadian medical graduates returned to academic practice in Canada, and 1 each from Thailand and Saudi Arabi returned to academic practice in their home country.

### Career Outcomes

Fifty-nine (59) of the 72 graduates (82%) secured careers in academic university institutions; there are 3 Professors, 11 Associate Professors, 43 Assistant Professors or Clinical Educators, and 2 Instructors. Eleven (11) of the 72 established careers in a large group medical practice, and 2 transitioned to industry. One graduate who returned to an academic institution is deceased (cancer).

There was a trend but no significant difference in the percentage of graduates in academia when cohort 1 was compared with cohort 2 (72% vs 90%, *p* = 0.073). IMG had a similar rate of having an academic career compared to US medical graduates (86% vs 72%, *p* = 0.197). There was no difference in the percentage of female graduates staying in academia compared with male graduates (94% vs 78%, *p* = 0.170).

### Academic Productivity during BMT-CT Fellowship Training

To allow for adequate lag time between completing the BMT CT fellowship and publication acceptance, fellows from July 1, 2022, to June 30, 2024, were not included in this analysis. Thus, the denominator for this comparison was 62 graduated fellows instead of the entire cohort of 72 graduates. As an aggregate of 62, the BMT CT fellows were scholarly active during their 12-month clinical fellowship, and 30 were listed on 46 peer-reviewed, original research publications. Twenty (20) fellows had at least one first-authored, peer-reviewed, original research publication; 10 other fellows were listed as co-authors on original research, peer-reviewed publications; and 2 other fellows published review articles.

To determine the possible impact of the planned mentoring program, we compared authorship in the 8-year period prior to structured mentoring (July 1, 2007–June 30, 2015) to authorship in the 7-year period that followed. Whereas the group of 23 fellows that graduated prior to structured mentoring contributed 8 publications, the 39 fellows in group that followed structured mentoring contributed 38 publications (*p* = 0.0001).

## Discussion

We report a 17-year, single-center review of the largest comprehensive and structured 12-month clinical BMT-CT fellowship program in the USA. Most (69%) of our fellows had graduated from international medical schools and studied in the USA on a non-immigrant J1 visa, yet only a small number (*n* = 6) returned to their home country. An individual’s visa status may be a possible reason that contributed to a decision to pursue additional sub-specialty education because the additional training facilitated transition to an immigrant visa [8, 9]. In addition, our 17-year experience revealed a consistent pattern: that a high rate of IMGs who completed a BMT-CT fellowship attained academic careers. This suggests that IMGs will provide a significant and steady supply of our academic subspecialty physician workforce in the coming decade.

While our Institution has DEI programs and policies to improve recruitment of underrepresented applicants, and our BMT-CT division faculty is comprised of diverse population, women and racially and ethnically minoritized fellows (Black and Latino) remained underrepresented throughout the 17-year of our BMT-CT fellowship program. This suggests that prioritizing a culture of diversity and inclusion in and of itself is not enough. We submit that new approaches are needed to address this under-representation by early engagement of fellows in the hematology-oncology training programs. Thus, one consideration could be to work within the American Society of Hematology (ASH) Medical Educators Institute (MEI) together with the ASTCT to create a network of paired hematology/oncology fellows who are interested in transplantation and cellular therapy with BMT-CT fellows/faculty mentors from within or outside of their primary institutions.

There is no comparable publication that reports in an unbiased manner of the demographic make-up, and career outcomes of adult BMT-CT fellows (Table 2). A multi-center effort reported the demographics and career outcomes of 59 questionnaire survey responders that trained in an adult BMT-CT fellowship program between 2012 and 2021 [10]. The number of trainees in this multi-center report is fewer than in the current report; importantly, the multi-center report represents a large unknown selection bias given that less than one-third of the pool of physicians contacted responded. It is possible that many of the 59 respondents represented those who were satisfied with their decision to pursue BMT-CT training and in academic institutions. A second multi-center publication [11] highlighted physician perceptions, recruitment, and retention to BMT-CT programs. The authors of this report sent an electronic survey to 947 ASTCT members in-training and those who were within the first 10 years of practice at the time of the survey. Just over 6% (*n* = 59) of those contacted responded of which half were female (*n* = 30). The low response rates and the other unknown selection biases intrinsic in these reports may have affected the generalizability of these reports. The current manuscript is unique in that it highlights the demographics, the impact of core curriculum changes on scholarly productivity, and career decisions in 73 BMT-CT fellows over a 17-year period in an unselected and unbiased manner.

**Table 2:** Comparison of fellowship programs

	Stanford	Benerjee et al Report	Sharma et al Report	ACGME report (2021–2022)	Horn et al report
Fellowship program	Adult BMT-CT	Adult/pediatric BMT	Adult/pediatric BMT	Adult Hem/Onc	Adult Hem/Onc
Number of fellows	*n* = 73 (inclusive)	*n* = 105 (out of 300–350)	*n* = 59 (out of 947)	*n* = 1970	*n* = 236 (out of 690)
Study method	Complete database	Survey	Survey	Report	Survey
*Population*					
Female	25%	51%	51%	45%	42%
IMG	70%	39%		36%	
URM	11%	14%	19%	11%	
*Outcomes*					
Academic career	82%	89%	Likely 76%		60%
Publications	52% (1 year)	88% (1 year)			64% (3 years)

*BMT-CT* blood and marrow transplantation-cellular therapy, *Hem/Onc* hematology/oncology, *IMG* international medical graduate, *URM* under-represented minorities

Arguably, some of our most noteworthy data is the high number of BMT-CT fellows in both cohort 1 and 2 that sought and maintained academic faculty positions. The rate of academic career of graduates from our program compared similarly or favorably to two survey results from other BMT fellowship programs [6, 7] (Table 2). In comparison, a survey complied from 28 hematology/oncology fellowship programs that are National Cancer Institute-Designated Cancer Centers and/or National Comprehensive Cancer Networks revealed that only 60% of graduated fellows opted for careers in academic practice [12]. While it is beyond the scope of our report to understand the factors that compel graduates of hematology/oncology fellowship programs to pursue academic careers or not, we believe it is in our purview to speculate about our graduates. When we established a core training curriculum in 2007, we created a standardized application process that included a discussion of career goals and paths with each applicant. Following each interview, the team of faculty interviewers submitted a scoresheet that included an evaluation of likelihood to stay in academic medicine, among other related assessments. Thus, we contend that a main reason a high number of our trainees sought academic careers reflected an initial selection bias. It has been reported that fellows seeking advanced sub-specialty training albeit in other disciplines associated with an increased likelihood of becoming an academic physician, and in having a higher research impact, and scholarly productivity [13]. In the field of hematology/oncology, it is hard to determine whether sub-specialty training in BMT-CT will provide additional motives or skills in choosing an academic career due to lack of details reports/information. However, the aggregate of information strongly supports that additional sub-specialty training in BMT-CT associated with a high likelihood of having an academic career in the field.

Effective mentorship is considered a key factor in shaping the career trajectory of a fellow [14]. In our program, we followed a paradigm that no two trainees had the same interests, needs, goals, strengths, areas for development, personality, or work-style. Following the introduction of a structured mentoring plan in 2015, each fellow was encouraged to select a primary and secondary faculty mentor from within the division. The scholarly productivity as assessed by peer-reviewed research publications authored by our fellows significantly increased in cohort 2. Coincidently, our division has more than doubled in faculty number, and it is possible that the larger pool of faculty helped contribute to increased scholarly productivity among our fellows.

In summary, individuals who seek additional post hematology-oncology fellowship training in BMT-CT represent a unique group of academically inclined trainees. As the educators in the field, we need to provide them a well-thought-out, structured clinical training program including planned mentorship in order to give them the skills for caring of patients with the highest quality and for continued academic contribution. We believe that our 17-year experience in such a program can be a blueprint for other institutes.

## Supplementary Information

Below is the link to the electronic supplementary material.

Supplementary file1 (DOC 92 KB)

Supplementary file2 (DOCX 15 KB)

## References

[1] Niederwieser D, Baldomero H, Atsuta Y et al (2019) One and Half Million Hematopoietic Stem Cell Transplants (HSCT). Dissemination, trends and potential to improve activity by telemedicine from the Worldwide Network for Blood and Marrow Transplantation (WBMT). Blood 134(supplement_1):2035

[2] Appelbaum F, Fay J, Herzig G et al (1995) American Society for Blood and Marrow Transplantation guidelines for training. Biol Blood Marrow Transplant 1(1):56 9118292

[3] Shpall E, Adkins D, Appelbaum F, Keating A, LeMaistre AF, Mangen K, Smith F, Council on Education and Standards, Task Force (2001) Guidelines for clinical centers and training. American Society for Blood and Marrow Transplantation guidelines for training. Biol Blood Marrow Transplant 7(10):577 12659107 10.1016/s1083-8791(01)70019-5

[4] Khan S, Juckett MB, Komanduri KV, Krishnan A, Burns LJ, American Society of Blood and Marrow Transplantation Committee on Education (2012) American Society of Blood and Marrow Transplantation guidelines for training in hematopoietic progenitor cell transplantation. Biol Blood Marrow Transplant 18(9):1322–1328 22522027 10.1016/j.bbmt.2012.04.007

[5] Jain T, Knight T, Alencar MC, Davis L, Rao K, Im A, Malone AK (2022) American Society for transplantation and cellular therapy guidelines for fellowship training in hematopoietic cell transplantation and immune effector cell therapy. Transplant Cell Ther 28(3):125–133 34954294 10.1016/j.jtct.2021.12.011

[6] FDA Statement from FDA Commissioner Scott Gottlieb, MD, and Peter Marks, MD, PhD, director of the Center for Biologics Evaluation and Research on New Policies to Advance Development of Safe and Effective Cell and Gene Therapies. Jan. 15, 2019. https://www.fda.gov/news-events/press-announcements/statement-fda-commissioner-scott-gottlieb-md-and-peter-marks-md-phd-director-center-biologics

[7] Aghajanian H, Rurik JG, Epstein JA (2022) CAR-based therapies: opportunities for immuno-medicine beyond cancer. Nat Metab 4(2):163–169 35228742 10.1038/s42255-022-00537-5 PMC9947862

[8] Boulmay B, Prowell T, Blaya B, Pietanza MC (2018) No decision is final: career planning and career transitions. Am Soc Clin Oncol Educ Book 38:881–886 30231402 10.1200/EDBK_200983

[9] Lewis AR, Choong GM, Cathcart-Rake E et al (2024) Preparing hematology/oncology fellows for success: implementing an annual career development and research retreat. J Cancer Educ 39(1):58–64 37848596 10.1007/s13187-023-02375-9

[10] Banerjee R, Kelkar AH, Durani U, Anagnostou T, Nishitani M, Mallhi K, Majhail NS, Logan AC (2023) Demographics, motivations, and experiences of participants in transplantation or cellular therapy fellowships. Transplant Cell Ther 29(5):394e1-394e7 10.1016/j.jtct.2023.03.011 36934994

[11] Sharma A, Czechowicz A, Mavers M, Chao N, DiPersi J, Reddy P, Perales M-A, Smith M (2024) Recruitment and retention of hematopoietic cell transplantation and cellular therapy physicians: a report from the ASTCT talent acquisition task force. Transplant Cell Ther 30(6):559–564 38608806 10.1016/j.jtct.2024.04.005 PMC11216222

[12] Horn L, Koehler E, Gilbert J, Johnson DH (2011) Factors associated with the career choices of hematology and medical oncology fellows trained at academic institutions in the United States. J Clin Oncol 29(29):932–3938 10.1200/JCO.2011.35.8663 PMC3189092 21911716

[13] Childers JT, Haff CW, Lack BT, Forbes JM, Jackson GR, Sabesan VJ (2024) Does an additional advanced degree influence career trajectory as a shoulder and elbow surgeon? J Shoulder Elbow Surg S1058–2746(24):00582–00592 10.1016/j.jse.2024.07.010 39168444

[14] Qureshy Z, Nair P, Vesely SK et al (2024) Evaluating the impact of a year-long external mentorship pilot program in classical hematology. Blood Adv 8(18):4833–4844 39087874 10.1182/bloodadvances.2024013218 PMC11416643

