# Supplementary material for: A 17-Year Experience of a Large Dedicated Fellowship in Blood and Marrow Transplantation and Cellular Therapy: A Blueprint for Modern Day Training Program
Source: J Cancer Educ. 2024 Nov 27;40(4):575–81. doi: 10.1007/s13187-024-02545-3 (PMC12310893; doi:10.1007/s13187-024-02545-3)
Supplement: Supplementary file 1 — Supplementary file1 (DOC 92 KB) [file 13187_2024_2545_MOESM1_ESM.doc]

**STANFORD UNIVERSITY SCHOOL OF MEDICINE**

**BLOOD AND MARROW TRANSPLANTATION & CELLULAR THERAPY (BMT-CT) CLINICAL FELLOWSHIP PROGRAM**

______________________________________________________________________

#### DESCRIPTION:

BMT-CT Fellowship

#### I. GOALS

1. To offer an intensive learning experience covering all aspects of clinical and laboratory hematopoietic cell transplantation and cellular therapy, and to prepare individuals for an academic career in this dynamic field.

2. To provide clinical and investigative training in adult hematopoietic cell transplantation including both autologous and allogeneic (related, unrelated and haploidentical) grafts using different conditioning (myeloablative, reduced intensity and non-myeloablative) regimens.

3. To provide additional clinical training in cellular therapies including but not limited to chimeric antigen receptor (CAR) T-cell therapy. The aim is to impart a detailed understanding of the principles, rationale, indications, mechanics, complications and results of bone marrow or peripheral progenitor cell transplantation and cellular therapy.

4. To encourage fellows to become integrated into a mentored clinical research project, where they will gain experience in data analysis and manuscript writing and submission.

#### II. OBJECTIVES

1. **PATIENT CARE**

**By the end of the fellowship:**

Objective 1: Demonstrate clinical skills of medical history and physical examination, with specific attention to complications related to immunosuppressed patients.

Objective 2: Demonstrate clinical skill in the diagnosis and management of graft-versus-host disease.

Objective 3: Demonstrate clinical skill in medical management of patients receiving high-dose chemotherapy and immunosuppressive agents.

Objective 4: Demonstrate competency in performing bone marrow aspirations and biopsies.

1. **MEDICAL KNOWLEDGE**

**By the end of this fellowship:**

Objective 1: Describe the history and evolution of hematopoietic cell transplantation (HCT) as a therapeutic modality.

Objective 2: Describe the use of HCT and cell therapy for the treatment of and the outcomes for the following diseases:

- Acute and chronic myeloid leukemias
- Acute and chronic lymphocytic leukemias
- Non-Hodgkin’s lymphoma
- Hodgkin’s disease
- Multiple Myeloma
- Myelodysplasia and myeloproliferative disorders
- Aplastic anemia

Objective 3: Describe the indications for autologous, allogeneic or matched unrelated transplantation and the differences in patient management, complications and outcomes between them.

Objective 4: Understand the diagnosis and management of complications of HCT including:

- Short- and long-term complications of preparative regimen chemotherapy and/or radiotherapy
- Graft versus host disease: diagnosis, pathophysiology, staging, prophylaxis and treatment
- Veno-occlusive disease: diagnosis, pathophysiology, prophylaxis and treatment
- Immunosuppression and infectious disease:
- Fungal and PCP prophylaxis
- Empiric antibiotic therapy for prolonged neutropenia
- Monitoring and treatment of CMV
- Selected ID, GI, pulmonary and reproductive complications

Objective 5: Describe the definition and biology of hematopoietic stem cells, the sources of hematopoietic cells, modifications of the HCT product and the use of growth factors. Part of the goal of this objective will be accomplished by rotating through the Stanford Cellular Therapeutics Laboratory (SCTT).

**C. PRACTICE-BASED LEARNING AND IMPROVEMENT**

Fellows will have the opportunity to apply medical literature, research and statistical methods, and data management technology for self-evaluation and improvement by regular attending the weekly new patient meeting, monthly morbidity and mortality conference and protocol meetings.

**By the end of the fellowship:**

Objective 1: Evaluate new patient, and formulate and research clinical questions through discuss during our regular clinical meetings. Access to the Stanford BMT database is available to assist with clinical decision making.

Objective 2: Select Cases for in depth research, discussion and presentation at the Hematopathology or New Patient conferences as appropriate.

**D. INTERPERSONAL AND COMMUNICATION SKILLS**

**By the end of the fellowship:**

Objective 1: Develop and demonstrate competence in effective and efficient communication with patients and their families.

Objective 2: Develop and demonstrate competence in effective and efficient communication with physician colleagues on the BMT service, physicians from other services, nursing and support staff. Since the BMT service involves interactions with multiple ancillary services including social work, dietary, physical therapy, blood bank, nurse coordinators as well as BMT Nurse Practitioners/Physician Assistants, communication is essential for the successful treatment of these patients.

**E. PROFESSIONALISM**

Fellows will have the opportunity to develop and demonstrate an increasing commitment to carrying out professional responsibilities and adherence to ethical principles. An effective therapeutic relationship with patients and families will be demonstrated through listening, narrative and nonverbal skills; education and counseling of patients and families.

**By the end of the fellowship:**

Objective 1: Demonstrate respect and compassionate use of medical skills for BMT patients. This includes the treatment of patients and families dealing with life-threatening illnesses undergoing toxic therapies.

Objective 2: Demonstrate respect and compassionate use of medical skills for BMT patients receiving comfort care.

**D. PROCEDURES**

1. Hematopoietic progenitor cell/Cellular therapy cell infusion: During the 12-month clinical rotation, each fellow is expected to perform 60-80 infusions of either hematopoietic progenitor cells or other cellular products. The fellows will learn the required and correct steps to ensure a precise and safe cell infusion procedure. The fellows will also learn how to manage infusion-related reaction and special situations such as broken bag (see attached Fellow training – Cell Infusions).

2. Bone marrow harvest: Fellows need to understand the principle and process of bone marrow harvest in the operation room. During the 12-month fellowship, each fellow is expected to preform TWO bone marrow harvests under the supervision of attending physician (see attached Physician training – Bone Marrow Harvest). Due to the irregular nature of bone marrow harvest schedule, the fellows might have the opportunity to do harvest either first or second half the year.

#### III. INSTRUCTIONAL METHODS

Ambulatory:

In the ambulatory setting, fellows will evaluate and manage BMT patients in BMT clinic and the Infusion Treatment Area.

Inpatient:

Fellows will receive BMT training primarily in the inpatient setting in the Compromised Host Unit as well as BMT patients in the ICU. The fellows will be part of a physician team including the Attending physician as well as residents in BMT, Hematology and Medical Oncology.

Schedule:

The educational content is provided in the following ways:

- Teaching sessions on the Inpatient Unit before and during inpatient rounds by the Attending physician and ancillary staffs
- Weekly didactic lectures on Friday at 8:00 AM
- Teaching sessions in the BMT Clinic
- New Patient conference on Tuesdays at 1:00 p.m.
- Participation in bone marrow aspirations/biopsies in the Infusion Treatment Area (Cancer Center)
- Reading Materials (see References below)

#### IV. SUPERVISION OF BMT-CT FELLOWS

BMT-CT Fellows will be under the direction and supervision of a BMT attending physician with respect to all inpatient and outpatient patient care. While fellows can help to formulate a treatment or management plan, all new changes in the care plan will be discussed fully with supervising attending physician. All documentation made by the fellows will be reviewed and co-signed by supervising attending physician.

The first THREE cell infusions at the start of fellowship need to be performed together with a supervising attending physician. After supervising attending acknowledge the ability of individual fellow to infuse cell product, future cell infusion can be carried out by fellow with a attending physician as standby. All bone marrow harvest will be done in operation room with a supervising attending physician regardless the experience of specific BMT-CT fellow.

Protocol defining common circumstances requiring faculty involvement

Fellows are encouraged to communicate with supervising attending physician any time they feel the need to discuss any matter relating to patient care.

The following are circumstances and events where the fellows or rotating residents **must** communicate with supervising attending physician:

1. ICU transfers, DNR or other end of life decisions
2. Encounters with any patient in emergency rooms
3. All new patient encounters in inpatient units
4. If requested to do so by other faculty attendings in any primary or specialty program
5. If specifically requested to do so by patients or family
6. If any error or unexpected serious adverse event is encountered at any time
7. If the fellow or resident is uncomfortable with carrying out any aspect of patient care for any reason
8. New onset cytokine release syndrome (CRS), immune effector cell-associated neurotoxicity syndrome (ICANS) and macrophage activation syndrome (MAS)
9. Onset of any severe adverse event (SAE) on any patient who is on clinical trial

#### V. EVALUATION

### ACGME Competencies

According to Accreditation Council of Graduate Medical Education (ACGME), training and evaluation must include the following competencies: Patient Care, Medical Knowledge, Practice – Based Learning and Improvement, Interpersonal and Communication Skills, Professionalism, and System-Based Practice.

**Patient Care** will be evaluated by global assessment of the attending physician and additional information from nursing staff. Procedures performed will be documented.

**Medical Knowledge** will be evaluated by global assessment of the attending physicians, and peer-reviewed chart audit.

**Practice-Based Learning and Improvement** will be evaluated by peer-reviewed chart audit.

**Interpersonal and Communication Skills** will be evaluated by global assessment of the attending physicians and additional information from nursing staff, other ancillary staff, patients and families.

**Professionalism** will be evaluated by global assessment of the attending physicians and additional information from nursing staff, other ancillary staff, patients and families.

**System-Based Practice** will be evaluated by global assessment of attending physicians, and peer-reviewed chart audit.

Evaluations are reviewed with the fellows for formal feedback. Face to face interaction between the attending physician and the fellow is the required method. At the midway point of the rotation, the fellow is encouraged to approach the attending to assess and discuss performance. In addition, ongoing feedback is provided related to fellows’ patient care responsibilities and activities. Fellows meet with the BMT Fellowship Director every three months to review their experience.

In addition, fellows provide feedback to the Attending Physicians and Fellowship Director regarding the rotation to add input on deficiencies in the experience.

#### There are three (3) planned evaluations:

**Initial Competency Assessment (at 3 months)**

Able to evaluate the suitability of new patients for transplantation or cellular therapy (score 1-9)

Able to present new patient evaluation in a precise and meaningful way at weekly new patient meeting (score 1-9)

Able to work as a team member efficiently in both inpatient and outpatient (ITA) setting caring of patients who undergo transplant or cellular therapy (score 1-9)

Able to interpret laboratory, pathology and radiology results pertinent to our patients. These may include but not limited to MRD, bone marrow biopsy, chest CT and CT/PET (score 1-9)

Able to work and communicate with other members of care team (APPs, nurses, pharmacists, social worker) effectively (score 1-9)

Have performed at least 3 cell infusions and able to administrate hematopoietic cell and cellular therapy cell infusion accurately and safely (yes or no)

Completion of cell therapy Risk Evaluation and Mitigation Strategy (REMS) program (yes or no)

**Semi-Annual Evaluation (at 6 months)**

Demonstrate understanding of the indications for autologous and allogeneic hematopoietic cell transplant (score 1-9)

Demonstrate understanding of the purpose of high dose therapy in autologous transplant and individual agents used (score 1-9)

Demonstrate understanding of different conditioning regimens in allogeneic transplant including myeloablative, reduced intensity and non-myeloablative (score 1-9)

Demonstrate understanding of different methods to collect hematopoietic progenitor cells and the differences between different graft sources (score 1-9)

Demonstrate understanding of human leukocyte antigen (HLA) typing and donor selection (score 1-9)

Demonstrate understanding of unique challenges in haploidentical and cord blood transplants (score 1-9)

Demonstrate understanding of the different presentations of acute graft-versus-host disease (GVHD) and its grading system (score 1-9)

Demonstrate understanding of the treatment principle of acute graft-versus-host disease (aGVHD) including novel therapies (score 1-9)

Demonstrate understanding of the different presentations of chronic graft-versus-host disease (cGVHD) in different organ systems (score 1-9)

Demonstrate understanding of design of chimeric antigen receptor (CAR) T-cells and their applications (score 1-9)

Demonstrate understanding of how to identify and manage complications associated with CART-cell therapy including cytokine release syndrome (CRS), immune effector cell-associated neurotoxicity syndrome (ICANS) and macrophage activation syndrome (MAS) – part of REMS program (score 1-9)

Demonstrate understanding of the management of infectious complications associated with hematopoietic cell transplants and cellular therapies including bacterial, fungal and viral agents (score 1-9)

Demonstrate understanding of immediate post transplant complications including sinusoidal obstruction syndrome (SOS) and diffuse alveolar hemorrhage (DAH) (score 1-9)

Demonstrate understanding of the management of post-transplant relapse in myeloid diseases (score 1-9)

Demonstrate understanding of the management of post-transplant relapse in lymphoid diseases (score 1-9)

Demonstrate ability to present and communicate in an accurate, organized and concise manner (score 1-9)

Demonstrate professionalism with respect, compassion, integrity, commitment and self-reflection at workplace (score 1-9)

Have performed a bone marrow harvest under the supervision of an attending physician (yes or no)

Register and take the board certification exam in either Hematology or Oncology administrated by American Board of Internal Medicine (ABIM) (yes or no)

Fellow strengths and areas for improvement (free text)

**Final Summative Evaluation**

Patient Care

Ability to evaluate new patients for transplantation or cellular therapy (score 1-9)

Ability to manage post transplant side effects/complications (score 1-9)

Ability to administrate hematopoietic cell and cellular therapy cell infusion accurately and safely (score 1-9)

Ability to evaluate donor for hematopoietic cell donation (score 1-9)

Ability to perform bone marrow harvest from healthy donor (score 1-9)

Ability to recognize and diagnose cytokine release syndrome (CRS), immune effector cell-associated neurotoxicity syndrome (ICANS) and macrophage activation syndrome (MAS) (score 1-9)

Medical Knowledge

Understanding the indications for autologous and allogeneic transplant (score 1-9)

Understanding the principle in the management of both acute and chronic GVHD (score 1-9)

Understanding the management of infectious complications in post transplant and cell therapy patients (score 1-9)

Ability to interpret laboratory, radiologic and pathologic result accurately (score 1-9)

Practice-Based Learning and Improvement

Ability to present and formulate treatment plan in weekly New patient meeting (score 1-9)

Ability to present and discuss cases in the monthly M & M meeting (score 1-9)

Ability to lead the daily discharge round in the two inpatient units (score 1-9)

Interpersonal and Communication Skills

Ability to communicate effectively with the patients and their family (score 1-9)

Ability to present and communicate effectively patient care issues with other members of the care team (score 1-9)

Ability to work with other members of care team (APPs, nurses, pharmacists, social worker) efficiently (score 1-9)

Ability to discuss cases with supervising attending and ask for guidance when needed (score 1-9)

Ability to finish documentation on time with clarity and accuracy (score 1-9)

Professionalism

Ability to treat patients, families, other team members with respect and compassion (score 1-9)

Ability to show integrity, commitment and self-reflection at workplace (score 1-9)

System-Based Practice

Ability to follow the guideline in the cell therapy Risk Evaluation and Mitigation Strategy (REMS) program (score 1-9)

Ability to follow the management guideline from the specialty society (American Society of Hematology, and American Society of Transplantation & Cellular Therapy) (score 1-9)

Monthly Evaluation Aggregate

Strength (free text)

Area for improvement (free text)

Other

Hematology Board Certified (yes or no)

Oncology Board Certified (yes or no)

During the entire time of fellowship, was this fellow ever subject to any disciplinary action, such as admonition, reprimand, suspension or termination? (yes or no)

To your knowledge, has the fellow ever been under investigation by any governmental or other legal body? (yes or no)

Have you ever observed the fellow to exhibit any behavior, drug, alcohol, or physical or mental impairment which has or reasonably could have been expected to interfere with this fellow’s ability to exercise the clinical duties in a safe and effective manner that is consistent with the prevailing standard of practice? (yes or no)

#### VI. SELECTED REFERENCES

This is a list of published articles that have critical and/or updated information on clinical practice and scientific research in the field of transplantation and cellular therapy. The BMT-CT fellows are encouraged to read these articles by themselves.

Scott et al. Myeloablative versus reduced-intensity hematopoietic cell transplantation for acute myeloid leukemia and myelodysplastic syndrome. J Clin Onc 2017, 35:1154

Spinner et al. Non-myeloablative TLI-ATG conditioning for allogeneic transplantation: mature follow-up from a large single-center cohort. Blood Adv 2019, 3:27

Anasetti et al. Peripheral-blood stem cell versus bone marrow from unrelated donors. NEJM 2012, 367:1487

# Meyer et al. Transplantation of donor grafts with defined ratio of conventional and regulatory T cells in HLA-matched recipients. JCI Insight 2019, 4:e127244

Yun et al. Clinical Relevance of Immunobiology in Umbilical Cord Blood Transplantation. J Clin Med 2019, 8:1968

# Zeiser at al. **Acute Graft-versus-Host Disease — Biologic Process, Prevention, and Therapy.** NEJM 2017, 377:2167

# Zeiser et al. Pathophysiology of Chronic Graft-versus-Host Disease and Therapeutic Targets. NEJM 2017, 377:2565

Luznik et al. High-dose, post-transplantation cyclophosphamide to promote graft-host tolerance after allogeneic hematopoietic stem cell transplantation. Immunol Res 2010, 47:65

# Nakasone et al. Risks and benefits of sex-mismatched hematopoietic cell transplantation differ according to conditioning strategy. Haematologica 2015, 100:1477

# Tiercy et al. How to select the best available related or unrelated donor of hematopoietic stem cells? Haematologica 2016, 101:680

Harris et al. International, Multicenter Standardization of Acute Graft-versus-Host Disease Clinical Data Collection: A Report from the Mount Sinai Acute GVHD International Consortium. BBMT 2016, 22:4

MacMillan et al. A refined risk score for acute graft-versus-host disease that predicts response to initial therapy, survival, and transplant-related mortality. BBMT 2015, 21:761

Hartwell et al. An early-biomarker algorithm predicts lethal graft-versus-host disease and survival. JCI Insight 2017, 2:e89798

# Luznik et al. HLA-haploidentical bone marrow transplantation for hematologic malignancies using nonmyeloablative conditioning and high-dose, post-transplantation cyclophosphamide. BBMT 2008, 14:641

Weng et al. Minimal Residual Disease Monitoring with High-Throughput Sequencing of T Cell Receptors in Cutaneous T Cell Lymphoma. Science Translational Medicine 2013, 5:214ra171

# Weng et al. Nonmyeloablative allogeneic transplantation achieves clinical and molecular remission in cutaneous T-cell lymphoma. Blood Advances 2020 4:4474

# Spiegel et al. CAR T cells with dual targeting of CD19 and CD22 in adult patients with recurrent or refractory B cell malignancies: a phase 1 trial. Nature Medicine 2021, 27:1419

# Kitko et al. National Institutes of Health Consensus Development Project on Criteria for Clinical Trials in Chronic Graft-versus-Host Disease: IIa. The 2020 Clinical Implementation and Early Diagnosis Working Group Report. BBMT 2015, 21:389

# Zeiser et al. Ruxolitinib for Glucocorticoid-Refractory Chronic Graft-versus-Host Disease. NEJM 2021, 385:228

# Cutler et al. Rituximab for steroid-refractory chronic graft-versus-host disease. Blood 2006, 108:756

# Sarantopoulos et al. How I treat refractory chronic graft-versus-host disease. Blood 2019, 133:1191

# Flowers et al. How we treat chronic graft-versus-host disease. Blood 2015, 125:606

Locke et al. Axicabtagene Ciloleucel as Second-Line Therapy for Large B-Cell Lymphoma. NEJM 2022, 386:640

Kamdar et al. Lisocabtagene maraleucel versus standard of care with salvage chemotherapy followed by autologous stem cell transplantation as second-line treatment in patients with relapsed or refractory large B-cell lymphoma (TRANSFORM): results from an interim analysis of an open-label, randomised, phase 3 trial. Lancet 2022, 399:2294

Scholler et al. Tumor immune contexture is a determinant of anti-CD19 CAR T cell efficacy in large B cell lymphoma. Nature Medicine 2022, 28:1872

Hourigan et al. Impact of Conditioning Intensity of AllogeneicTransplantation for Acute Myeloid Leukemia WithGenomic Evidence of Residual Disease. J Clin Onc 2019, 38:1273

Goldstone et al. In adults with standard-risk acute lymphoblastic leukemia, the greatest benefit isachieved from a matched sibling allogeneic transplantation in first completeremission, and an autologous transplantation is less effective than conventionalconsolidation/maintenance chemotherapy in all patients: final results of theInternational ALL Trial (MRC UKALL XII/ECOG E2993). Blood 2008, 111:1827

**VII. SUMMARY TABLE**

| **Hands-on Learning** | | |
| --- | --- | --- |
| Inpatient Rotation | 3.5-4 months | Learning objectives:   1. To understand the different conditioning regimens. 2. To understand the different hematopoietic cell graft sources and their clinical application. 3. To learn how to manage immediate post-transplant toxicity. 4. To learn about the common infectious complications, including bacterial and fungal infections. 5. To be familiar with the presentation of acute graft-versus-host disease (GVHD) and the management. 6. To learn how to manage critically ill post-transplant patients. 7. To be familiar with the immediate side effects/complications after chimeric antigen receptor (CAR) T-cells therapy, including CRS and neurotoxicity. 8. To learn how to manage CRS and neurotoxicity. 9. To learn how to manage flares of chronic GVHD. 10. To perform all cell infusions, including CART cells. |
| Outpatient Rotation | 2 months | Learning objectives:   1. To manage the side effects/complication of post autologous transplant patients. 2. To learn how to manage neutropenic fever in the outpatient setting. 3. To learn how to diagnose acute graft-versus-host disease (GVHD) and its management. 4. To learn how to manage early relapsed disease after transplant. 5. To learn how to monitor patients with chimeric antigen receptor (CAR) T-cells therapy. 6. To manage central line-related issues. 7. To learn how to manage viral reactivation, especially CMV, EBV, HHV-6 and BK virus. 8. To learn how to evaluate post-transplant patients with acute illness. 9. To be familiar with non-myeloablative allogeneic transplant and the unique nature of this outpatient regimen. |
| Clinic Rotation | 4 months | Learning objectives:   1. To learn about indications for transplantation in different diseases. 2. To learn how to determine the appropriateness of referred patients to be considered for transplant. 3. To determine the best transplant regimen for individual patients. 4. To learn different presentations of chronic GVHD of each organ system. 5. To learn the principles of managing chronic GVHD. 6. To learn how to manage relapsed disease after transplantation. 7. To be familiar with critical issues of long-term health of post-transplant patients. |
| Research Rotation | 2.5 months | Learning objectives:   1. To learn how to conduct clinical trials. 2. To learn about the regulatory aspects of clinical trials (i.e., IRB, consenting, SRC). 3. To be familiar with common statistical tools for clinical research. 4. To learn how to prepare poster presentations and write manuscripts. |
| **Procedure** | | |
| Hematopoietic progenitor cell/Cellular therapy cell Infusion | Each fellow is expected to perform 60-80 infusions of either hematopoietic progenitor cells or other cellular products. The fellows will learn the required and correct steps to ensure a precise and safe cell infusion procedure. The fellows will also learn how to manage infusion-related reaction and special situations, such as a broken bag. | |
| Bone marrow Harvest | Each fellow is expected to perform 2-3 bone marrow harvests with supervising faculty member(s). The fellows will learn the correct technique of bone marrow aspiration in the operation room and how to collect them in a sterile manner using special close collection system. | |
| **Practice-based Learning** | | |
| New patient meeting | weekly | Learning objectives:   1. To present and discuss new patient evaluation. 2. To discuss difficult or interesting cases in both the inpatient and outpatient service. |
| Morbidity & Mortality conferences (transplant and cell therapy) | monthly | Learning objectives:   1. To evaluate cases of morbidity and mortality follow transplant or cell therapy and perform a root cause analysis of the clinical outcomes of the patients |
| Protocol meetings (transplant and cell therapy) | monthly | Learning objectives:   1. To discuss the science and protocol for emerging or active clinical trials in BMT-CT |
| **Knowledge-based Learning** | | |
| Didactic lecture | weekly | The weekly lecture covers four major areas:   1. Principles related to BMT and cellular therapy including conditioning regimen, GVHD, infectious complications, management of toxicity and relapse, HLA, and donor selection. 2. Utility of BMT-CT and current practice in different diseases. 3. Clinical trial related topics including the how to develop IIT and practical aspects of conducting and managing a clinical trial. 4. Research within the institute |
| Journal club | bi-weekly | The journal club is conducted by fellows with paper selected by faculty member and under one/two faculty member’s supervision at each session. |
| Reading list |  | A list of important publications in the field of BMT and cellular therapy selected by the entire faculty members. The fellows are expected to read all the recommended publications by the end of 12-month program. |
| **Mentoring and Networking** | | |
| One-on-one mentoring |  | Fellowship director will meet with individual fellow every 3 months for monitoring progress, adjusting the rotations based on clinical interest, career advice and job search. |
| Research mentoring |  | Each fellow is encouraged to identify a faculty member as a research mentor at the beginning of the fellowship. This research mentor will work with individual fellow to develop and conduct a research project, to prepare the abstract for national meeting and write the manuscript at the end of the research project. |
| National meetings (ASH and ASTCT) | annually | National meetings in the fields of Hematology, transplant, and cellular therapy, which offer the opportunity to present their research, learn about novel science and relevant clinical updates, and network with colleagues |
| Stanford BMT-Cell Therapy Symposium | bi-annually | Two-days meeting to highlight the important updates on the science and practice in the field of BMT-CT |
