# Supplementary material for: A 17-Year Experience of a Large Dedicated Fellowship in Blood and Marrow Transplantation and Cellular Therapy: A Blueprint for Modern Day Training Program
Source: J Cancer Educ. 2024 Nov 27;40(4):575–81. doi: 10.1007/s13187-024-02545-3 (PMC12310893; doi:10.1007/s13187-024-02545-3)
Supplement: Supplementary file 2 — Supplementary file2 (DOCX 15 KB) [file 13187_2024_2545_MOESM2_ESM.docx]

**Structured Mentoring Plan – Overview**

1. Within 8 weeks of starting fellowship the trainee will meet one-on-one with the fellowship director. The theme of the first meeting is to encourage a self-assessment, ask yourself:

What are my main goals?

What type of work would I like to be doing?

What kind of environment would I like to be in?

Where would I like to be in an organization?

What is important to me in a career?

How do you value work life balance?

What is my personality and work-style?

What is my time-line for achieving my career goals?

Do you believe your time-line is accurate?

1. Within 12 weeks of starting, a second meeting to review self-assessment replies. Begin to prioritize an approach on how to achieve goals, set time lines for applications for post fellowship employment. Encourage finding a primary and secondary faculty mentor within the division, and if uncertain suggestions can be offered based on self-assessment responses. There is no a priori intended distribution of the trainees among the faculty members, rather suggest discussion with as many faculty members as possible, and based on interests and personalities mentorship will be established.
2. Discuss and routine check ins about J1 Visa extensions, transition to dual intent visas.
3. Discuss contributing to a research publication – this is not a requirement as it is predominantly a clinical training fellowship. Explain that this requires working after hours and on one’s free time during weekends and holidays, just like a real faculty job.
4. Goal to establish bi-monthly meetings with primary mentor.
5. Choose between 1 of 2 annual three-day scientific and career development research retreats geared to pre- and post-doctoral fellows that are sponsored by faculty within the Division of Hematology and Oncology, or Stanford Immunology and with participation by BMT CT faculty. These retreats are at the Asilomar Conference Center located in Pacific Grove, about 90 minutes from Stanford University.
6. Choose between attendance at the American Society of Hematology (ASH) or Tandem meeting (ASTCT and CIBMTR).
